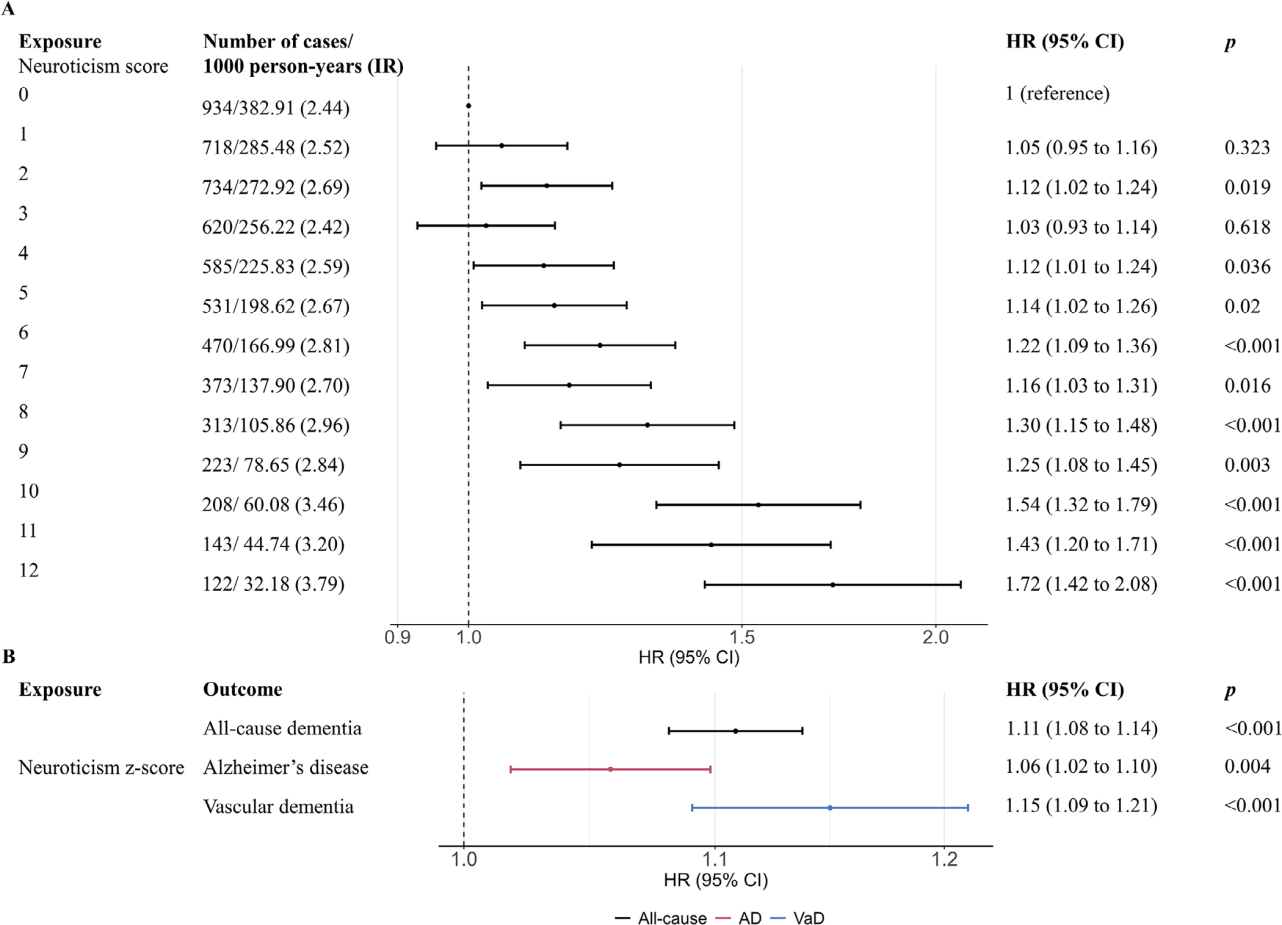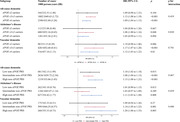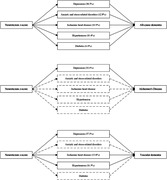# Neuroticism and risk of all‐cause dementia, Alzheimer’s disease, and vascular dementia: exploring modification by genetic risk and mediation by vascular and mental health

**DOI:** 10.1002/alz.087545

**Published:** 2025-01-09

**Authors:** Yaqing Gao, Najaf Amin, Cornelia M Van Duijn, Thomas J Littlejohns

**Affiliations:** ^1^ University of Oxford, Oxford United Kingdom; ^2^ University of Oxford, Oxford, Headington United Kingdom

## Abstract

**Background:**

Neuroticism is a personality trait that is typically stable across the life course. Higher neuroticism has been linked to a higher risk of developing dementia in studies primarily consisting of small sample sizes. There is a need to explore the association in larger populations, whilst uncertainty remains regarding the mechanisms driving the associations.

**Method:**

We selected 174,164 participants aged 60 years or older who were dementia‐free at baseline from the UK Biobank cohort. Cox proportional‐hazards models were used to assess the association between standardized neuroticism score (z‐score) with risk of all‐cause dementia, Alzheimer’s disease (AD), and vascular dementia (VaD). Models were adjusted for age, sex, ethnicity, Townsend‐deprivation index, education, smoking status, alcohol intake, and body mass index. We investigated effect modification by genetic predisposition for dementia and mediation through mental and vascular health conditions. The association between neuroticism and dementia‐related neuroimaging outcomes was investigated in a sub‐sample who underwent an imaging assessment (n = 39,459).

**Result:**

Over a median follow‐up of 13.5 years, 5,974, 2,741, and 1,364 participants developed all‐cause dementia, AD, and VaD, respectively. Each unit increase in neuroticism z‐score corresponded to an 11% (hazard ratio [HR] 1.11, 95% Confidence Interval [CI] 1.08‐1.14), 6% (HR = 1.06, 95% CI 1.02‐1.10]), and 15% (HR = 1.15, 95% 1.09‐1.21) higher risk of all‐cause dementia, AD, and VaD, respectively. The associations remained similar regardless of genetic predisposition for dementia risk. Depression, anxiety and stress‐related disorders, ischaemic heart disease, and hypertension mediated 38.5%, 12.8%, 10.9% and 10.4% of the total association between neuroticism and all‐cause dementia, with similar mediation proportions observed for dementia subtypes. Neuroticism was significantly associated with a lower volume of total grey matter and a higher volume of white matter hyperintensities, a marker of cerebrovascular health.

**Conclusion:**

Neuroticism is associated with an increased risk of dementia, particularly VaD and cerebrovascular pathology, regardless of genetic risk. Mental and vascular health may mediate this association. Neuroticism could represent a marker for dementia risk stratification and addressing the increased burden of mental and vascular health conditions in individuals with higher neuroticism could, if causal, ultimately help prevent or delay the onset of dementia.